# Investigation of alpha smooth muscle actin changes in the liver of cholestatic rats following the consumption of L-theanine

**DOI:** 10.1016/j.toxrep.2025.102193

**Published:** 2025-12-18

**Authors:** Mobina Daneshnia, Pejman Mortazavi, Mahsa Ale-Ebrahim, Razieh Hosseini

**Affiliations:** a Faculty of Veterinary Medicine, SR.C., Islamic Azad University, Tehran, Iran; b Department of Veterinary Pathobiology, SR.C., Islamic Azad University, Tehran, Iran; c Department of Physiology, TMS.C., Islamic Azad University, Tehran, Iran; d Department of Veterinary Basic Sciences, SR.C., Islamic Azad University, Tehran, Iran

**Keywords:** α-SMA, BDL, Cholestasis, L-Theanine, Liver fibrosis

## Abstract

Cholestatic liver disease represents a major global health threat, resulting in significant morbidity and mortality. Cholestasis can be induced in laboratory animals using Bile Duct Ligation (BDL) technique. Activated Hepatic Stellate Cells (aHSCs) express Alpha Smooth Muscle Actin (α-SMA), which is correlated with experimental liver fibrogenesis. The *Camellia sinensis* plant produces L-Theanine, an amino acid (AA), in its roots. This research endeavored to conduct a comprehensive assessment of the anti-fibrotic effects of L-Theanine alongside α-SMA changes in the liver of cholestatic rats. Rats were classified into eight experimental groups, each consisting of five animals, including; (1) normal control group, (2) BDL control group, (3–5) healthy experimental groups, 6–8) BDL + L-Theanine groups. L-Theanine solution (100, 200 or 400 mg kg^−1^) was administered to the animals by Intragastric gavage (once a day) for 30 successive days. BDL significantly elevated the enzymatic activity of Gamma-glutamyl transferase (GGT), Alanine aminotransferase (ALT), Alkaline phosphatase (ALP), Aspartate aminotransferase (AST) and elevated the amount of total bilirubin. These biochemical alterations were ameliorated when L-Theanine was administered. Masson`s Trichrome and Immunohistochemical (IHC) staining revealed that BDL expanded the collagen deposition and α-SMA expression in hepatic tissue. Administration of L-Theanine, remarkably alleviated these alterations. L-Theanine attenuates hepatic fibrosis through decreasing the production of α-SMA.

## Highlights

• L-Theanine alleviated hepatic injuries.
• L-Theanine diminished the expression of α-SMA in hyperplastic bile duct cells.
• L-Theanine decreased collagen accumulation in cholestasis.
• L-theanine reduced the increase of biochemical parameters in cholestasis.

## Graphical Abstract

**Figure:**
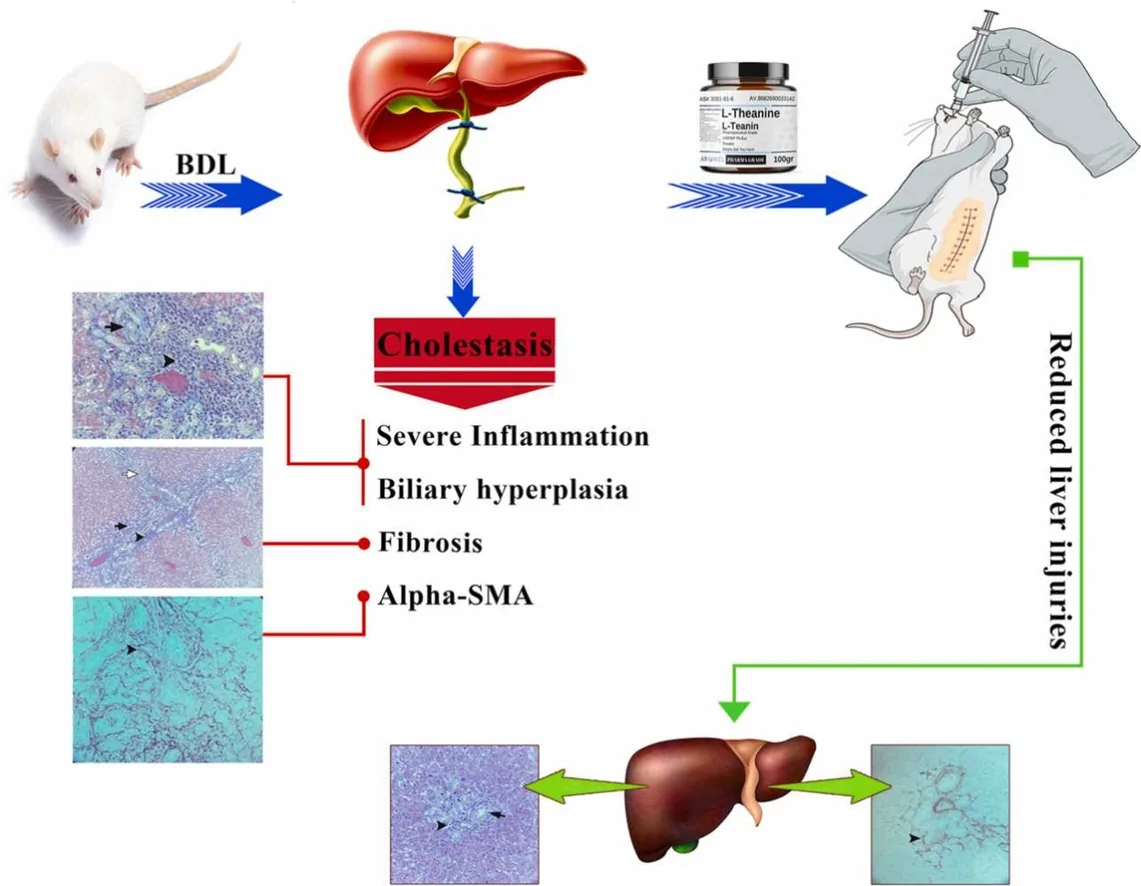

## 1 Introduction

Annually, liver disorders result in approximately two million mortalities across the globe and currently, cirrhosis ranks as the eleventh leading cause of death universally [1]. Impairment in the circulation of bile constituents is called cholestasis. Cholestasis is generally classified into intrahepatic and extrahepatic cholestasis [2], [3]. One of the conventional experimental models which is conducted to induce cholestasis and liver fibrosis is BDL. The underlying mechanisms of inflammation and fibrosis can be perceived by BDL experimental model [4]. Upon cholestasis, the impaired excretion of bile salts results in their toxic accumulation within hepatocytes which leads to excessive collagen deposition [5], [6]. The apoptotic effects of bile acids may be mediated through multiple pathways, including Fas death receptor activation and the initiation of oxidative stress, ultimately resulting in mitochondrial permeability transition (MPT) and mitochondrial dysfunction [7], [8], [9]. Oxidative stress induces Kupffer cells to synthesize tumor necrosis factor alpha (TNF-α), which in turn exacerbates cellular inflammation, apoptosis, and lipid peroxidation [10], [11]. When chronic liver damage occurs, hepatocytes go through apoptosis and secrete factors that attract fibroblasts, Kupffer cells and bone marrow macrophages to the injured areas. Afterwards these cells produce TGF-β1, which can trigger the activation of HSCs into myofibroblasts [12]. Upon activation, HSCs exhibit a strong cytoplasmic immunoreactivity for α-SMA. α-SMA, an isoform of actin, serves as a distinctive indicator for the differentiation of smooth muscle cells [13]. α-SMA expression in liver mesenchymal cells is perceived to be vigorously correlated with liver fibrosis in humans and rats [14]. Long-term cholestatic conditions can result in hepatic fibrosis, cirrhosis and liver failure which can be fatal in severe cases and emphasizes the necessity of liver transplantation [15]. L-Theanine is an exceptional AA that is primarily accumulated within the foliage of the *Camellia sinensis* plant. Glutamic acid and ethylamine serve as substrates for the enzyme theanine synthetase, which catalyzes the synthesis of this non-protein AA.

L-Theanine is massively utilized as a safe supplement in drinks and food products. L-Theanine is simply available, rapidly absorbed through the intestine and metabolized by the liver [16], [17]. Numerous investigations have confirmed that L-Theanine can diminish oxidative impairments by decreasing lipid peroxidation, reducing reactive oxygen species (ROS) production and increasing glutathione (GSH) concentration. Moreover, L-Theanine has the potential to improve the antioxidant capability of hepatocytes which is accomplished through the restoration of antioxidant enzyme activities, including superoxide dismutase (SOD), GSH, and catalase (CAT), alongside the prevention of malondialdehyde (MDA) production in hepatic injury [18], [19]. It has been reported that L-Theanine remarkably up-regulated Bcl-2 while concurrently down-regulating Bax and other apoptotic indicators [20]. L-Theanine as a hepatoprotective compound, prevented hepatocyte injury against ethanol toxicity. The reduction of mitochondrial membrane potential (MMP) and the release of cytochrome C from mitochondria were both suppressed by L-Theanine [19]. Further research indicated that L-theanine suppressed the inflammatory response due to oxidative stress, which involves the enhancement of TNF-α and Interleukin-1 beta (IL-1β) in the serum along with cyclooxygenase-2 (COX-2) and inducible nitric oxide synthase (iNOS) expression in the liver [21]. A significant neuroprotective effect is exerted by L-Theanine through its attachment to glutamate receptors. It also protects neurons from death with its antioxidant and anti-inflammatory properties [22], [23]. Scientific evidence suggests that L-theanine might prevent the peroxidation of low-density lipoprotein (LDL), hence the risk of atherosclerosis [24].

Over the last twenty years, the incidence and prevalence of cholestasis has risen worldwide, making it a significant public health concern [25]. Since hepatic fibrosis ranks among the leading causes of mortality universally, finding an effective treatment for it is highly relevant [26].

At the present time, no research has investigated the role of L-Theanine in cholestatic liver fibrosis. This study was endeavored to investigate α-SMA alterations in the liver of cholestatic rats following the consumption of L-Theanine.

## 2 Material and methods

### 2.1 Animal models

Forty mature male *Wistar* rats (each weighed approximately 220–250 g with the age of 9–11 weeks) were sourced from the Pasteur Institute of Iran. The animal models were maintained at the animal research laboratory, faculty of veterinary medicine, science and research branch university in Tehran. The animals were kept in routine cages under a 12 h photoperiod, within an environment kept at a stable thermal condition of 22 ± 2 °C and humidity level of 40–55 %. Rats were fed with commercial pellets and had unlimited access to tap water for 7 days prior to experimental exposure. Animal welfare and experimental procedures were performed precisely following the appropriate guidelines and regulations. Furthermore, the animals were treated in accordance with the protocols sanctioned by the Ethical Committee of Islamic Azad University, Science and Research Branch, Tehran, Iran (Approval date: 2024–05–11, Approval no: IR.IAU.SRB.REC.1403.097)

### 2.2 Experimental design

After adaptation to laboratory conditions, rats were accidentally classified into eight experimental groups of five animals each and the classification was as follows:

The normal control group (intact) and BDL control group were orally administered 1 cc of sterile distilled water once a day (via intragastric gavage) for 30 successive days. The healthy experimental groups and other BDL groups were orally administered (100, 200, or 400 mg kg^−1^ BW respectively) three different doses of L-Theanine solution (dissolved in distilled water) via gavage (once daily) for 30 consecutive days. L-theanine administration was initiated post-BDL surgery (day 1) and continued for 30 successive days. A conventional method was utilized to accomplish BDL [4]. After the rats were administered intramuscular and intraperitoneal anesthesia with ketamine (80 mg/kg BW) and xylazine (20 mg/kg BW), a midline laparotomy of approximately 3 cm in length was performed, using surgical scalpel blade No.11 and the connective tissue was dissected with a scissor. We elevated the liver with a moisturized cotton swab and the bile duct was revealed by moving the intestine downward. Two 4–0 silk sutures were inserted at two points (one was placed in the beginning of common bile duct and the other was located before the entrance of pancreatic duct). The abdominal cavity was flushed with saline solution and the organs were returned to their normal location. The peritoneum and muscle layers were sutured using 4–0 Vicryl, while the skin was sutured using 4–0 silk. A heating pad maintained at 37 °C was utilized throughout the surgical procedure to prevent hypothermia in anesthetized rats. Then the rats were placed in cages heated by an infrared lamp for recovery; until they became fully awake and active.

### 2.3 Chemicals

L-Theanine (CAS: 3081–61–6, HPLC 98.0–102.0 %) was purchased from Aromel Kimya Medikal A.S (Istanbul, Turkey). Commercial assay kits for ALT, AST, ALP, GGT, Total bilirubin and Total protein were all purchased from Delta Darman Part Company, Iran. Ketamine 10 % and Xylazine 2 % were acquired from Alfasan Company, Netherlands. All other chemicals were of the best quality available in the market.

### 2.4 Biochemical assays

Following a 30-day investigation period, the animals underwent an 8 h fasting interval and then euthanized by intraperitoneal (IP) injection of an overdose of ketamine-xylazine combination. Blood samples were carried out by cardiac puncture and were gathered in tubes containing gel and clot activators. Following a twenty-min break for coagulation, the serum was isolated from blood samples via centrifugation at 5000 rpm for a 10 min duration. Levels of serum ALT, AST, ALP, GGT, Total bilirubin and Total protein were determined using BT-1500 Autoanalyzer, Italy with commercial assay kits. It should be noted that at the beginning and at the conclusion of the experimental phase, the body weights of all experimental groups were assessed, and the changes were recorded. On the ultimate day of the experiment, after complete liver dissection, the liver weights were recorded and then a section of the liver was instantly fixed in 10 % buffered formaldehyde solution (over a 48-h duration) for further histopathological investigations.

### 2.5 Histopathological assessment

Formalin-fixed samples were dehydrated by passing through gradient ethanol, followed by xylene clearing and embedding in paraffin blocks. Microtome sectioning of paraffin-embedded tissue blocks yielded 4 µm thick sections, which were then dyed with hematoxylin and eosin (H&E) and Masson`s trichrome for subsequent light microscopic analysis. The histopathological scores which were used to transform the qualitative data into quantitative data in order to use it in the Kruskal-Wallis test were classified as follows (at 400 × magnifications in 10 randomly selected sites):

Necrosis; 0 = absent, 1 = local injury< 25 %, 2 = local injury 25–50 %, 3 = massive, yet local injury, 4 = massive injury.

Infiltration of inflammatory cells; 0 = absent, 1 = local< 25 %, 2 = local 25–50 %, 3 = massive, yet local, 4 = massive.

Bile duct hyperplasia; 0 = no visible ductal hyperplasia, 1 = ductal hyperplasia< 25 % of individual lobules, 2 = ductal hyperplasia between 25–50 % of individual lobules, 3 = massive, yet local ductal hyperplasia, 4 = massive ductal hyperplasia.

Connective tissue; 0 = none, 1 = elevated collagen deposition with no septum, 2 = partial septum; 3 = complete and narrow septum, 4 = complete and dense septum [27], [28].

All sections were independently scored by two pathologists blinded to group allocation, with discrepancies resolved through consultation.

### 2.6 Immunohistochemistry

Immunohistochemical study was conducted by achieving 5 µm sections from paraffin-embedded tissue blocks and locating them on microscope slides. The sections underwent deparaffinization, washing in phosphate-buffered saline (PBS), and antigen retrieval. Following the administration of blocking buffer (Serum 10 % + bovine serum albumin (BSA)), the tissue samples were allowed to incubate with an Anti-Alpha Smooth Muscle Actin antibody (Abcam, USA-Cat:51451). To observe the activity of peroxidase, the tissue samples were soaked in diaminobenzidine (DAB), resulting in brown-colored products (The criterion for Alpha-SMA positivity is nuclei brown reaction) [28]. It should be noted that the negative control was performed with all the above steps on a liver tissue without using a primary antibody. Positive control for anti-α-SMA antibody was accomplished using tissue samples of human breast carcinoma.

Alpha-SMA alterations were assessed in the following manner:

Score 0 (absent), Score 1 (slight), Score 2 (intermediate), Score 3 (significant), corresponding to less than 3 %, 3–33 %, 34–66 %, and greater than 66 % of cells exhibiting α-SMA positivity, respectively [28].

Immunohistochemical staining was quantitatively analyzed using ImageJ software to evaluate staining intensity and the percentage of positive cells, supplementing the semi-quantitative scoring system.

### 2.7 Statistical analysis

GraphPad Prism 8.0 software was utilized for conducting quantitative assessment. The statistical examination of categorical data was carried out using two-way Analysis of Variance (ANOVA) and Kruskal-Wallis test. P-value below 0.05 was regarded as a statistically meaningful difference.

## 3 Results

### 3.1 Biochemical indicators of liver function

As visualized in (Fig. 1.A-E), BDL remarkably elevated the enzymatic activities of ALP, GGT, ALT, AST and the amount of total bilirubin, whereas L-Theanine itself did not modify the amount of serum total protein (Fig. 1.F). Compared to the BDL control group, BDL+L-Theanine groups significantly alleviated these biochemical alterations dose-dependently (P < 0.0001). As demonstrated in (Fig. 1), treatment of the healthy experimental groups with L-Theanine at doses of 100, 200, 400 mg kg^−1^ BW respectively, did not result in remarkable changes compared to the intact group. The peak reduction of these enzymatic activities as well as the amount of total bilirubin was obtained in the treatment with L-Theanine at the dose of 400 mg kg^−1^ BW compared to the BDL control group (P < 0.0001). These biochemical findings were completely consistent with the results of our histopathological assessments.

**Fig. 1:**
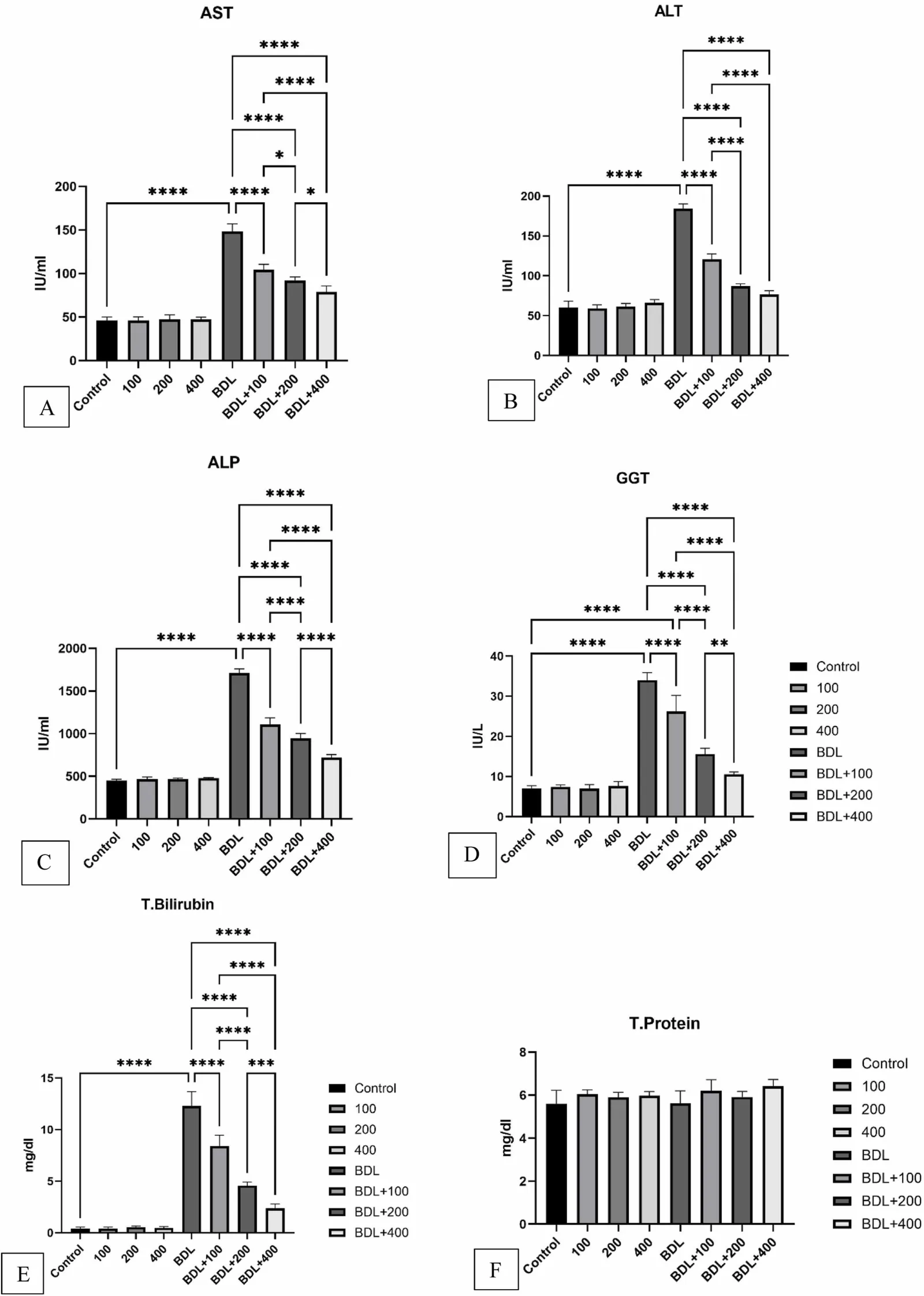
Effects of L-Theanine on serum biochemical indexes in BDL-induced cholestasis in Wistar rats. The normal control (intact) and BDL control groups recieved 1 cc of sterile distilled water. Experimental groups (healthy and BDL) were administered L-theanine at doses of 100, 200, or 400 mg/kg BW. Data are expressed as mean±SD (****P < 0.0001, ***P < 0.001, ** P < 0.01 and *P < 0.05) (n = 5). A- AST, B- ALT, C- ALP, D- GGT, E- Total bilirubin, F- Total protein.

### 3.2 Gross pathological alterations

L-Theanine consumption at doses of 100, 200, 400 mg/kg BW in healthy experimental groups did not lead to significant macroscopic changes in comparison with normal control group. The gross appearance of livers in BDL control group were enlarged, yellowish (as well as visceral surfaces of the other organs) with round edges. These macroscopic changes were slightly attenuated by L-Theanine consumption at the dose of 400 mg kg^−1^ BW.

As illustrated in (Fig. 2), BDL tremendously increased the absolute liver weights (P < 0.0001) in comparison to intact group. However, L-Theanine consumption alleviated these alterations dose-dependently (significant at the dose of 400 mg/kg BW).

**Fig. 2:**
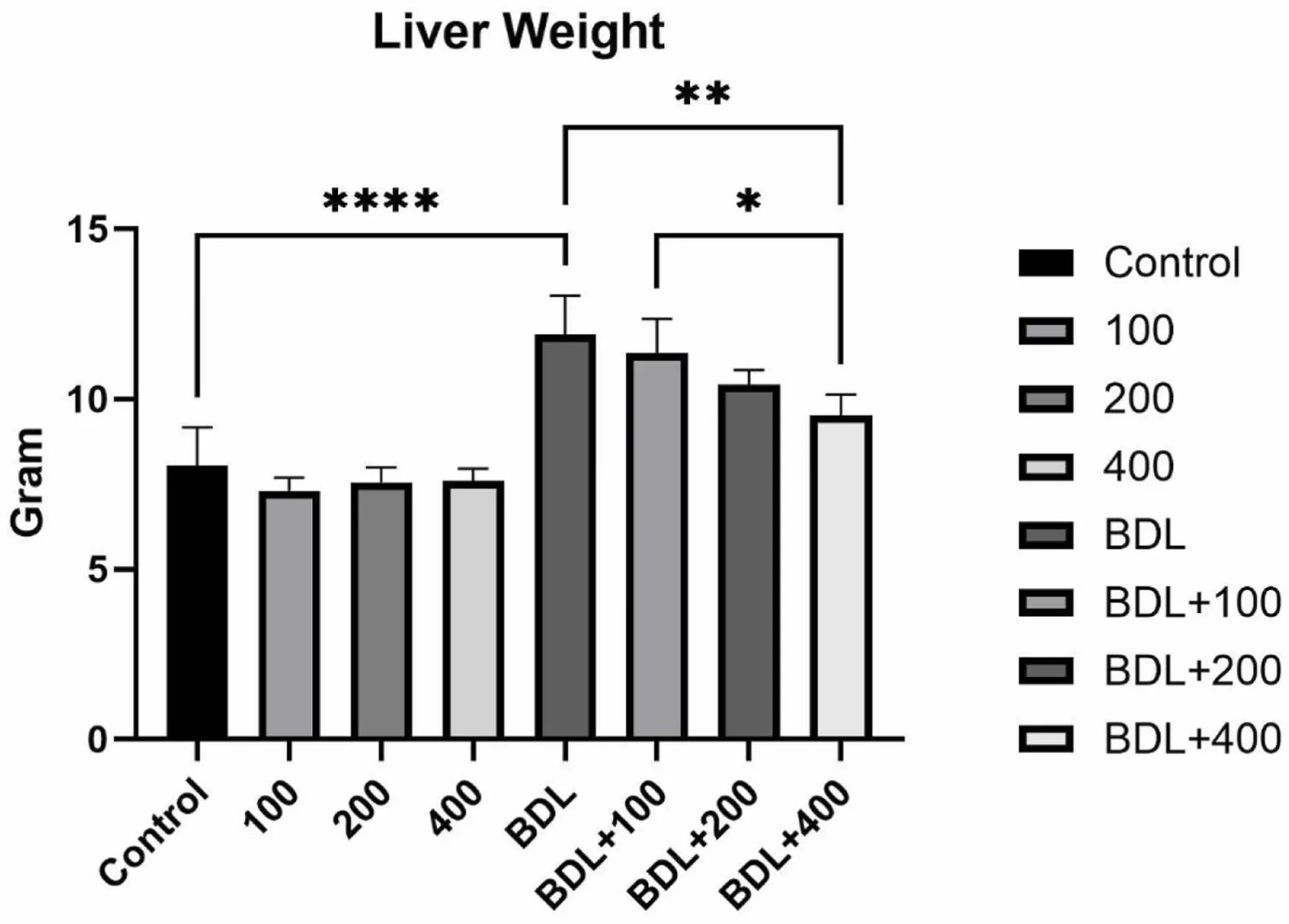
Liver weight changes in the studied groups. Controls (healthy and BDL) received 1 cc of sterile distilled water; experimental groups (healthy and BDL) were treated with L-theanine (100, 200, or 400 mg/kg BW). Data are expressed as mean±SD (****P < 0.0001, **P < 0.01, * P < 0.05) (n = 5).

### 3.3 Histopathological changes

As demonstrated in (Fig. 3), the pathological scores of the BDL control group were markedly higher than the normal control group. L-Theanine treatment significantly relieved the pathological scores dose-dependently (P < 0.0001).

**Fig. 3:**
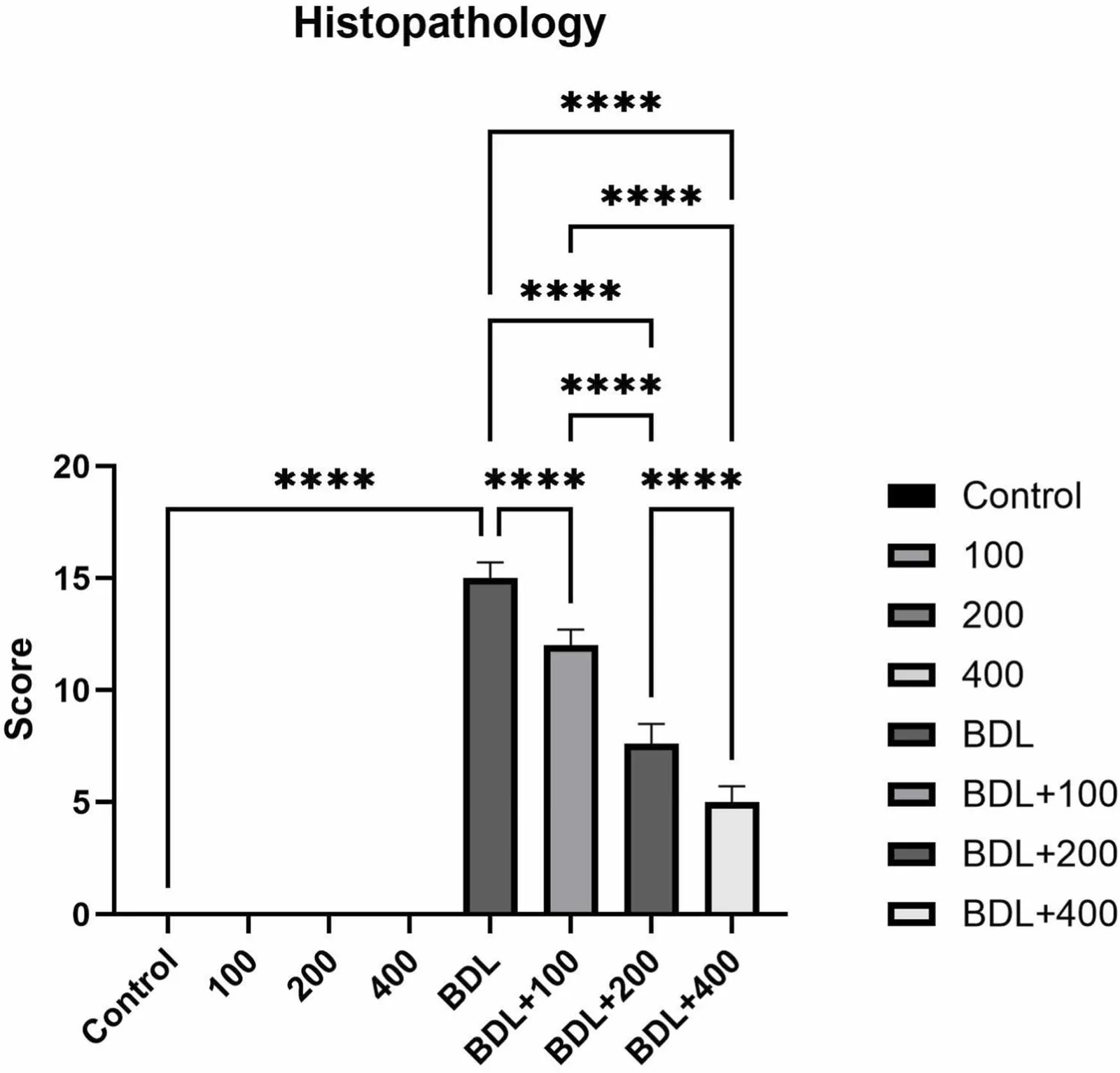
Histopathological injuries in the studied groups. Pathological scores in the BDL control group were markedly higher than those in the normal control group, while L-theanine treatment (100, 200, or 400 mg/kg BW) significantly alleviated these scores (****P < 0.0001) (n = 5).

Histopathological examination using Masson`s trichrome staining, demonstrated that the treatment of the healthy experimental groups with L-Theanine at doses of 100,200,400 mg/kg BW, did not result in any tissue damage in the liver in comparison with the intact group. In the healthy experimental groups, no deposition of fibrotic fibers, infiltration of inflammatory cells, and hepatocellular injury were visualized, and the morphological structure of the liver was completely identical to the intact group with intact sinusoids, hepatocytes and portal tract (Fig. 4.A-D). Meanwhile, the formation of a complete septum of collagen connective tissue in addition to other cholestatic liver damage parameters, including severe infiltration of mononuclear inflammatory cells and severe biliary hyperplasia were visualized in the BDL control group (Fig. 4.E, F). As presented in (Fig. 4.G-I), treatment of the BDL groups with L-Theanine considerably diminished liver fibrosis and tissue injury dose-dependently. The results of histopathological evaluations showed that L-Theanine at a dose of 400 mg/kg was drastically effective in reducing fibrosis, biliary hyperplasia, hepatocellular necrosis and inflammation (Fig. 4.I).

**Fig. 4:**
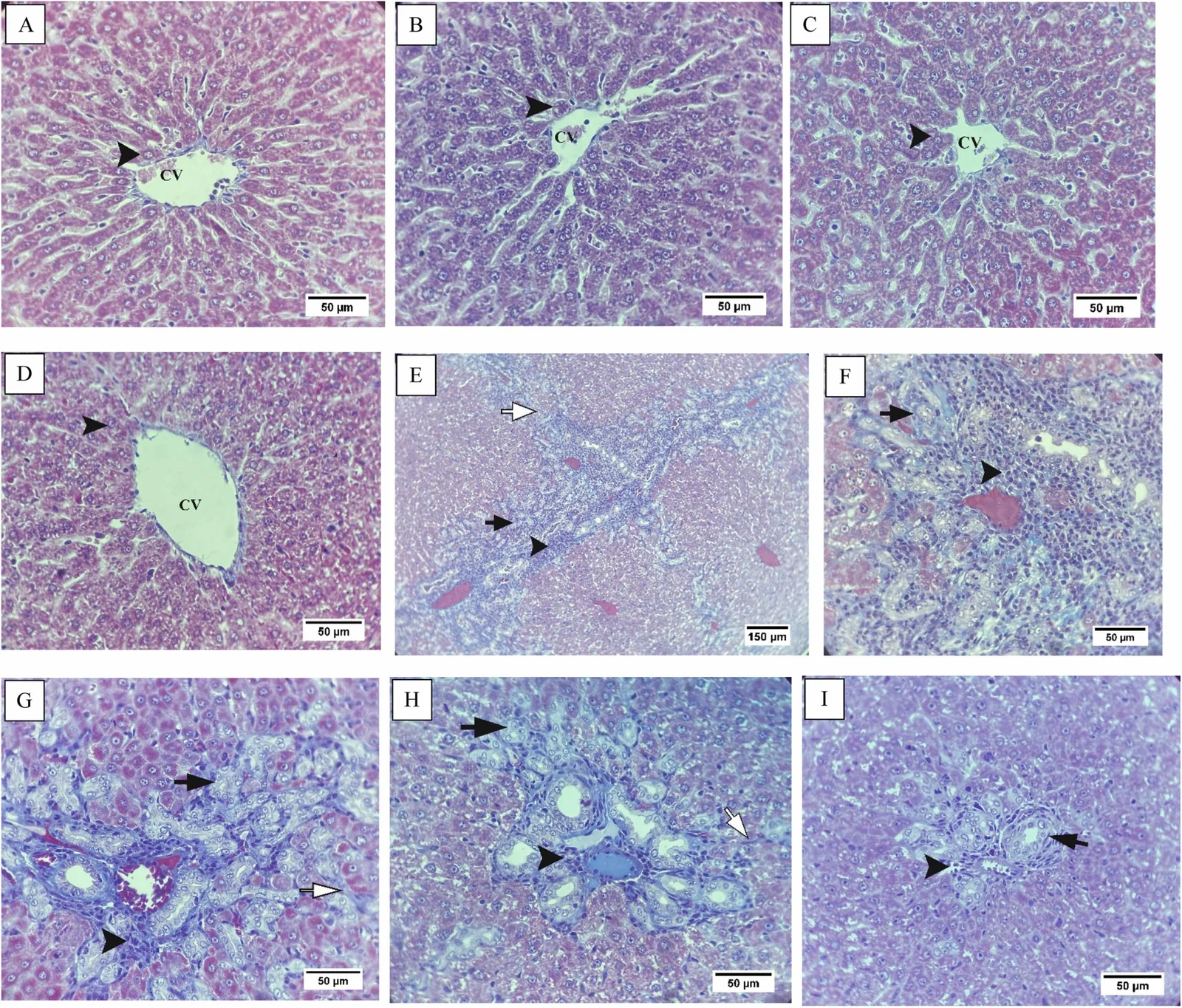
Representative Masson`s trichrome-stained histological photomicrographs of liver tissues following respective treatments. 30 days after the experiment, Masson`s trichrome staining was carried out. Histopathological changes in rat liver sections due to L-theanine administration (×400 magnification(Fig. 4_A,B,C,D,F,G,H,I_), × 100 magnification (Fig. 4_E_)). A- Normal control group (intact), B-D- Healthy experimental groups + L-Theanine (100,200,400 mg/kg BW respectively), E-F- BDL control group, G-I- BDL + L-Theanine (100,200,400 mg/kg BW respectively). No histopathological alterations were visualized in liver tissues of normal control group (A) and healthy experimental groups (B-D) and the central vein (CV) and intact hepatocytes (Arrowhead) were clearly observed as well as regular morphological architecture of the liver tissue. In BDL control group (E-F), Infiltration of connective tissue as complete septum (white arrow), excessive biliary hyperplasia (black arrow) and severe infiltration of mononuclear inflammatory cells (arrowhead) were observed. All these injuries were considerably alleviated by L-Theanine consumption (G-I) (significant at the dose of 400 mg/kg BW (I)).

α-SMA is predominantly localized within myocytes and smooth muscle cells surrounding the blood vessels. In cholestatic groups, alongside the mentioned areas, it is visualized in myofibroblasts within fibrotic regions.

In the present research immunohistochemical staining was accomplished in order to determine the expression levels of α-SMA fibrogenic factor within the hepatic tissue, following the consumption of L-Theanine. As evidenced in (Fig. 5.A-D), α-SMA expression was only positive around the central vein endothelium in both the normal control group and the healthy experimental groups that received L-Theanine. In contrast, α-SMA expression was notably elevated in the hepatocytes and hyperplastic bile duct cells (in addition to the mentioned areas) in the BDL control group. According to data presented in (Figs. 5,6), Treatment of the BDL groups with L-Theanine diminished the expression of α-SMA in the hepatic tissue dose-dependently. The BDL group which was administered L-Theanine at a dose of 400 mg/kg BW, showed a considerable reduction in α-SMA expression within hyperplastic bile duct cells.

**Fig. 5:**
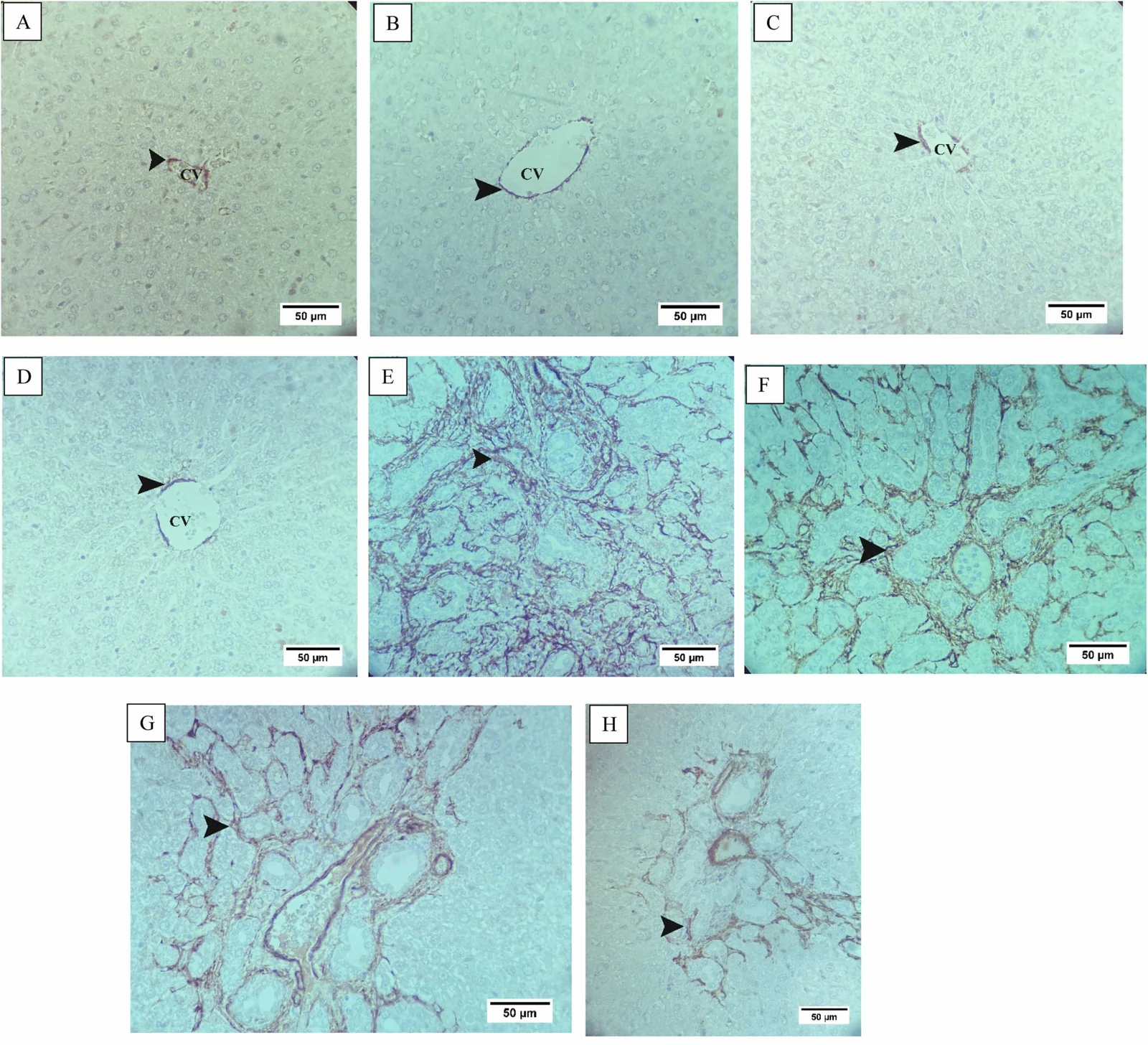
Immunohistochemical staining was carried out to evaluate the expression of α-SMA in the liver tissue sections in studied groups (IHC,400 ×). A- Normal control group (Intact) and B-D- Healthy experimental groups + L-Theanine (100,200,400 mg/kg BW respectively): negative expression of α-SMA was observed in hepatocytes located around the central vein and other hepatocytes. Expression was only seen around the central vein endothelium (arrowhead). E-Cross-section of liver tissue in BDL control group, excessive expression of α-SMA was observed in hepatocytes (arrowhead). F- BDL + 100 mg/kg L-Theanine, Significant expression of α-SMA was seen in hyperplastic bile duct epithelium and hepatocytes (arrowhead). G- BDL + 200 mg/kg L-Theanine, remarkable expression of α-SMA was visualized in hyperplastic bile duct epithelium (arrowhead). H- BDL + 400 mg/kg BW L-Theanine, Mild expression of α-SMA was observed in hyperplastic bile duct epithelium (arrowhead).

**Fig. 6:**
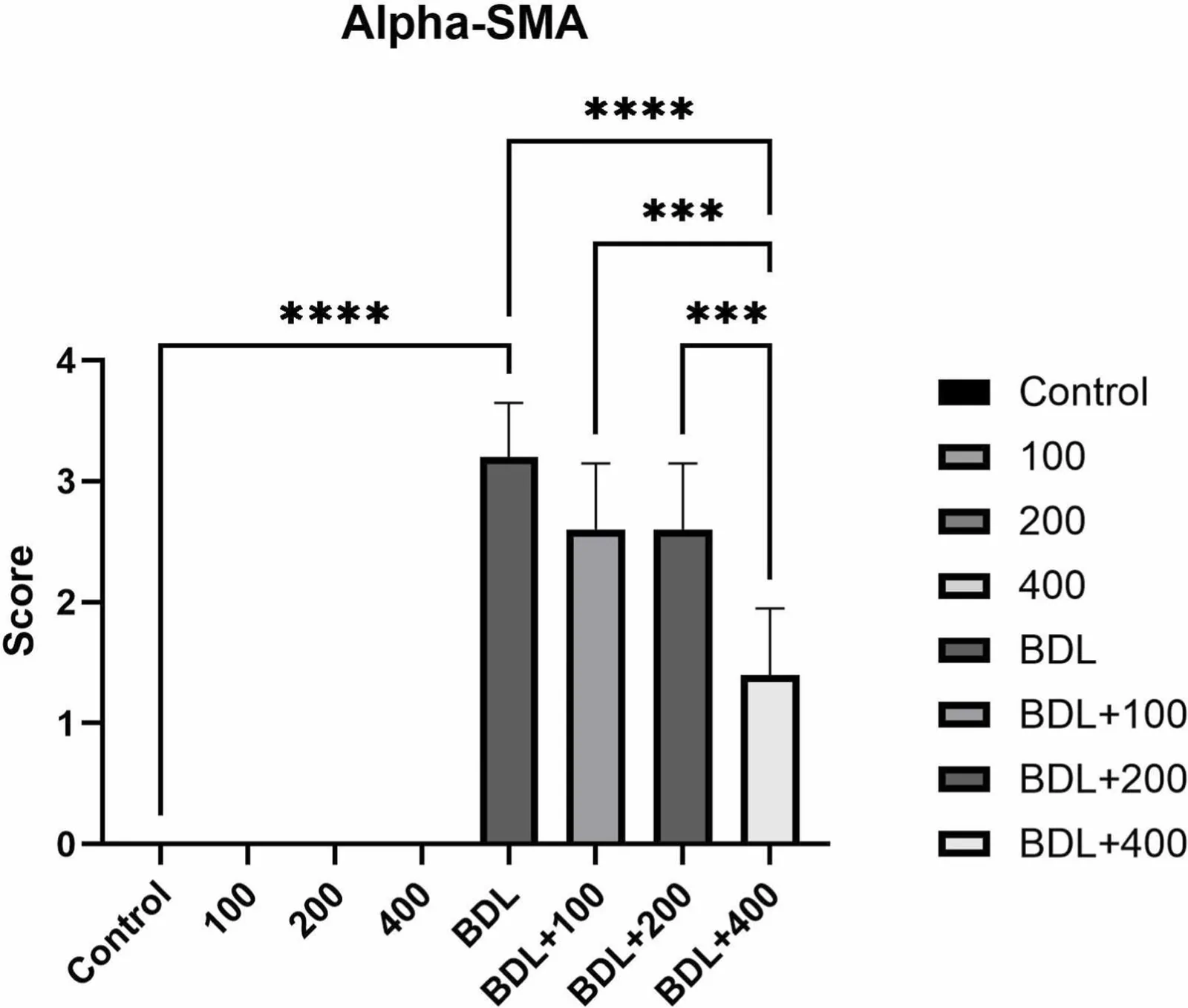
Statistical analysis of α-SMA expression in hepatocytes across studied groups. BDL control showed elevated α-SMA levels compared with normal control, while L-theanine (100, 200, 400 mg/kg BW) significantly reduced α-SMA expression, with 400 mg/kg BW being the most effective dose (****P < 0.0001, ***P < 0.001) (n = 5).

## 4 Discussion

Cholestasis triggers the retention of bile acids within hepatocytes, this induces a cascade of pathological events including hepatocellular injury and inflammation which can result in bile duct fibrosis and cirrhosis [29]. Liver fibrosis represents a progressive pathological condition which is distinguished by an accumulation of extracellular matrix (ECM) proteins in damaged liver tissues and can lead to structural and functional impairment of the organ [28], [30]. Today, it has been proven that liver fibrosis can be reversed, even in its final stages, once the underlying cause is eliminated [31]. This issue emphasizes the necessity of discovering effective treatments to control and inhibit liver fibrosis.

L-Theanine exhibits an appropriate safety profile, and its LD50 in rats exceeds 5000 mg kg^−1^. Furthermore, it has shown neither mutagenic nor carcinogenic properties in animal models [32], [33]. In the present investigation, BDL was carried out in order to induce cholestasis. Based on the histopathological assessments of the present study, BDL significantly induced liver fibrosis, which was accomplished as the formation of complete septums of connective tissue (Fig. 4.E_)._ On the contrary, these injuries were remarkably relieved by L-Theanine consumption (Fig. 4.I). Other cholestatic liver damage parameters including severe infiltration of inflammatory cells (especially lymphocytes), severe biliary hyperplasia and hepatocellular necrosis were also attenuated by L-Theanine consumption dose-dependently (significant at the dose of 400 mg/kg BW) (Fig. 4.I).

Previous studies have suggested that L-theanine may diminish oxidative impairments [19] and exert anti-inflammatory effects by suppressing the hepatic TNF-α/Nuclear factor kappa B (NF-κB) signaling pathway [34]. Furthermore, L-Theanine has been proved to decrease the secretion of IL-1β and IL-6 [35]. While our study did not directly assess these mechanisms, the observed improvements in fibrosis and α-SMA expression are consistent with such reports.

The histopathological and biochemical assessment of the current investigation revealed no considerable change or adverse effect in the healthy experimental groups in comparison with intact group. In addition, no cognitive disorder was observed in the mentioned groups, and there was no record of mortality. Nagai et al. [36] demonstrated that L-Theanine represents hepatoprotective properties against acute liver injury induced by Doxorubicin (DOX). This protective effect is associated with the inhibition of intrinsic apoptotic signaling pathways, specifically those mediated by caspase-3.

Various studies showed that in response to liver injury, HSCs become activated and exhibit a remarkable α-SMA immunoreactivity. aHSCs can be identified by their expression of α-SMA [13]. As it can be seen in (Fig. 5.A-D)_,_ α-SMA expression was primarily observed around the central vein (CV) endothelium in the intact group and healthy L-Theanine-treated groups. However, in the BDL control rats, α-SMA expression was considerably elevated within hepatocytes and bile duct cells in addition to the mentioned areas (Fig. 5.E). Najjar et al. [37] showed that biliary obstruction in rats activates the TGF-β1/Smad2/α-SMA signaling pathway after 2 weeks, which offers mechanistic context for the fibrogenic changes observed in our model, although this pathway was not directly investigated in the present study. Dong et al. [38] revealed that the excessive α-SMA expression in HSCs represents a key factor in the pathogenesis of liver fibrosis and might serve as an exceptional prognostic indicator for its progression. Previous studies have reported that L-theanine can inhibit NF-κB signaling, leading to a considerable down-regulation of pro-inflammatory and pro-fibrotic mediators, including TGF-β [39].

Our findings represented that treatment of the BDL groups with L-Theanine, diminished α-SMA expression in the liver dose-dependently. The BDL group which was administered L-Theanine at a dose of 400 mg/kg BW, exhibited a remarkable abatement of α-SMA expression in bile duct cells (Fig. 5.H).

Several types of liver and biliary diseases can precipitate an elevation in the enzymatic activity of ALP. This might be due to increased enzyme production, dissolution of cell membranes by sequestered bile acids and bleb release following cell injury [40]. When cholestasis is the cause of elevation in the enzymatic activity of ALP, the concentration of total bilirubin and bile acids may increase simultaneously [41], which concurs with the findings of this investigation. Acute hepatic injury can result in a sudden increase in serum activity of GGT, possibly attributed to the release of the cellular membrane fragments that bind to GGT [40]. As reported by Bulle and Leonard, increase in the production and release of GGT and subsequent enhancement of its enzymatic activity occurs with cholestasis and biliary hyperplasia [42], [43]. ALT and AST are leakage enzymes that are mainly found in the cytoplasm. Hypoxia, metabolic alterations, bacterial toxins, inflammation, liver neoplasia, and a large number of toxic chemicals can cause hepatocyte damage, resulting in ALT leakage [44].

The findings of the current investigation demonstrated that the BDL control group exhibited a notable enhancement in the enzymatic activity of AST, ALT, GGT, ALP and the amount of Total Bilirubin in comparison with the intact group. Treatment of the BDL rats with L-Theanine caused a notable reduction in the enzymatic activity of AST, ALT, GGT, ALP and total bilirubin levels. The maximum reduction was obtained at a dose of 400 mg/kg BW, which is also in accordance with the results of our histopathological assessments.

This study presents compelling evidence that L-Theanine consumption alleviated liver fibrosis caused by BDL. The therapeutic effect was likely attributed to the observed reduction in α-SMA expression. L-Theanine emerges as an exceptional therapeutic candidate for the prevention and management of cholestatic liver disease.

## Limitations of the study

This study has limitations that should be considered. Although histological, immunohistochemical, trichrome, and biochemical analyses were performed in a cohort of 40 Wistar rats, mechanistic investigations were not included. Specifically, oxidative stress markers (e.g., MDA, GSH), fibrosis-related signaling pathways (e.g., TGF-β1/Smad, NF-κB), and collagen metabolism indicators were not assessed, which limits insight into the molecular basis of L-theanine’s effects. Future studies incorporating broader mechanistic and molecular approaches are warranted to strengthen the translational relevance of these findings.

## Ethical Compliance Statement

All animal experiments complied with ARRIVE guidelines and followed the Animals (Scientific Procedures) Act 1986, EU Directive 2010/63, and the NIH Guide for the Care and Use of Laboratory Animals.

## Funding

This research did not receive any specific grant from funding agencies in the public, commercial, or not-for-profit sectors*.*

## CRediT authorship contribution statement

**Pejman Mortazavi:** Writing – review & editing, Visualization, Validation, Supervision, Software, Project administration, Methodology, Formal analysis, Data curation, Conceptualization. **Mobina Daneshnia:** Writing – original draft, Visualization, Resources, Project administration, Investigation, Funding acquisition, Data curation, Conceptualization. **Mahsa Ale-Ebrahim:** Writing – review & editing, Validation, Supervision, Methodology, Investigation, Data curation. **Razieh Hosseini:** Writing – review & editing, Methodology, Investigation, Data curation.

## Declaration of Competing Interest

The authors declare that they have no known competing financial interests or personal relationships that could have appeared to influence the work reported in this paper.

## Data Availability

Data will be made available on request.

## Glossary

AA: Amino Acid
α-SMA: Alpha Smooth Muscle Actin
AST: Aspartate aminotransferase
ALP: Alkaline phosphatase
ALT: Alanine aminotransferase
aHSCs: activated Hepatic Stellate Cells
BDL: Bile Duct Ligation
BW: Body Weight
BSA: Bovine Serum Albumin
CV: Central Vein
CAT: Catalase
Cox-2: Cyclooxygenase-2
DAB: Diaminobenzidine
DOX: Doxorubicin
DW: Distilled Water
ECM: Extracellular Matrix
GGT: Gamma-glutamyl transferase
GSH: Glutathione
HSC: Hepatic Stellate Cell
H&E: Hematoxylin and Eosin
IHC: Immunohistochemistry
IL-1β: Interleukin-1 beta
IL-6: Interleukin-6
IP: Intraperitoneal
iNOS: inducible nitric oxide synthase
LDL: Low Density Lipoprotein
LD50: Lethal dose 50
MPT: Mitochondrial Permeability Transition
MDA: Malondialdehyde
MMP: Mitochondrial Membrane Potential
NF-κB: Nuclear Factor Kappa B
PBS: Phosphate-buffered saline
ROS: Reactive Oxygen Species
SOD: Super Oxid Dismutase
TGF-β1: Transforming Growth Factor Beta 1
TNF-α: Tumor Necrosis Factor Alpha
